# Hints to Young Practitioners—No. I

**Published:** 1872-07

**Authors:** 

**Affiliations:** Savannah, Georgia


### HINTS TO YOUNG PRACTITIONERS—No. I.

BY SENEX.

It has been said that he is the best physician who administers the right remedy, at the right time, and in the right degree.

It would be curious to see a Dispensatory for 1972; I believe it will be a smaller volume than the Wood & Bache of to-day.

Our facetious brother, Oliver Wendell Holmes, who delights to have a laugh at his profession sometimes, summons up a man of the year 18072, and asks a number of questions; here are a few of them:

"Has any serious accident happened to the planet in the last thousand years?"

"What is the present form of religious belief?"

"What fuel is in use since coal gave out?" etc., etc.

He suggests the asking of a great many other questions; amongst them, it would be entertaining, if not profitable, to inquire:

"How many articles of the Materia Medica of 1872 remain besides quinine, opium, mercury, ipecac, chloroform, iodide of potassium, and iron?"

"When were the essential causes of malarial and zymotic diseases discovered?"

"What diseases, besides those of the zymotic class, are now considered essentially self-limited?"

"When did the profession make it penal to resort to the use of the speculum uteri on insufficient cause, and when was poking uterine sounds and sponge tents into the uterus on all occasions forbidden by statute?"

"How long did the homœopathic humbug last, and what humbug succeeded it?"

"What placebos succeeded arsenic, witch-hazel, and carbolic acid, in the treatment of wounds and bruises?"

"When was the procreation of the race regulated by law?"

"Has the lancet been permanently cast aside?"

Having in a measure satisfied our curiosity about things future, let us cast a retroverted eye to some of the curious things of the past.

A hundred years ago, a German surgeon gained great repute by his success in the treatment of gunshot wounds. His fame spread over the world, and his secret was sought eagerly by the profession. When discovered, the following was the formula of this vaunted vulnerary: "Take two puppies before their eyes have opened, drown them in olive oil, add a pint of earth-worms and a bottle of wine, and simmer all together for two hours."

A century before, when the merry monarch of England was smitten with apoplexy, his doctors bled him, and gave a distillation of human skulls, and thus foreshadowing homœopathy.

Coming to later times, we are met by the celebrated aphorism of Armsbnry—"Bleeding is the right arm of medicine, and mercury the left."

Southwood Smith, Physician to the London Fever Hospital, bled his patients mercilessly. Dr. Horner, in his memoir of Dr. Physic, mentions that he was bled eleven times by Dr. Dewees, in an attack of yellow fever; and now we are told by Warren Stone, in an address before the Academy of Medicine of New-York, that he is afraid to give even a dose of castor oil in that disease—confining his treatment almost to recumbency in bed, and good nursing, with some stimulation.

It is needless to discuss the question whether the abandonment of the lancet, or its almost abandonment, be due to change of type in disease, as ably contended for by Sir Thomas Watson, Dr. Nott, and others, or to improved views of therapeutics and pathology, as advocated by Bennett and those of his class. Certain it is, that the disturbing and depressing modes of treatment find little favor with the best physicians of the present day, and the knowledge when "not to do" is reckoned no small part of true wisdom. On the other hand, we are not to fold our arms in the presence of the enemy. Sir William Gull, in an address before the Clinical Society of London, thus expresses himself: "We believe that God's will is that we should be wise and active; and when we say, 'Thy will be done' (and I am sure our profession is not behind any in saying it), it is not that, like Moslems, we shall sit down and let the supposed inevitable result pass over us. As students and practitioners of medicine, our interpretation of the Divine prayer is in an active sense, and should excite us to every exertion. In therapeutics, the greater often depends upon the less. It has been said that the Duke of Wellington overcame his great antagonist by attending to the shoes of his soldiers. We often see great purposes frustrated by neglect of trifles: a patient brought through his fever, and dying of a bed-sore."

SAVANNAH, GEORGIA, July 20th, 1872.

